# Celebrating our heritage: the *Jornal Brasileiro de Pneumologia* and the *Sociedade Brasileira de Pneumologia e Tisiologia*

**DOI:** 10.36416/1806-3756/e20250303

**Published:** 2025-11-19

**Authors:** Ricardo de Amorim Corrêa

**Affiliations:** 1. Faculdade de Medicina, Universidade Federal de Minas Gerais - UFMG - Belo Horizonte (MG) Brasil.; 2. Sociedade Brasileira de Pneumologia e Tisiologia - SBPT - Brasília (DF) Brasil. (President, 2025-2026)

At times like these, it is essential to recall important moments in history. In October 1974, the Brazilian Society of Pulmonology was founded. Fifty years ago, in 1975, a visionary group of Brazilian pulmonologists, led by the *Sociedade Brasileira de Pneumologia*, recognized the urgent need for a specialized scientific platform to share research and clinical experiences focused on respiratory diseases. On October 18, 1978, the Brazilian Society of Pulmonology and the Brazilian Federation of Tuberculosis and Respiratory Diseases Societies merged into a single institution. The new society was renamed the Brazilian Society of Pulmonology and Phthisiology and adopted the founding date of the Brazilian Federation of Tuberculosis and Respiratory Diseases Societies, which is 1937.It is, in fact, the sum of the experience, successes, struggles, and greatness of two entities that, in the past, brought together lung specialists from across our country. From that commitment to excellence was born the *Jornal de Pneumologia* (JP)-a publication destined to become the leading scientific voice of pulmonary medicine in Brazil and a reference for the Portuguese-speaking world.^1^

From its earliest issues, the JP reflected the most pressing public health challenges in Brazil, such as the relentless fight against tuberculosis, which was the main cause of respiratory morbidity and mortality at the time. The journal played a pioneering role in disseminating regional research, clinical advances, and public health strategies, contributing directly to educational efforts and the improvement of respiratory care across the country.^2^

As Brazilian pulmonology-along with the SBPT and its committees-progressed, so too did the JP. In the 1980s and 1990s, it expanded its scope to include a wide array of topics-ranging from asthma and COPD to the increasing burden of smoking-related diseases, interstitial lung conditions, and advances in diagnostic and therapeutic techniques. The creation of new editorial sections and the embrace of thematic issues demonstrated the journal’s agility and ongoing commitment to the respiratory community.^1^ ^,^ ^2^

At the dawn of the new millennium, the JP decisively moved toward internationalization. In 2004, on the basis of a survey of SBPT members, it was decided that the journal would be renamed the *Jornal Brasileiro de Pneumologia* (JBP).^1^ The adoption of an electronic submission system, publication in Portuguese and English, and the journal’s inclusion in major scientific databases such as SciELO (since 2002), PubMed/MEDLINE (since 2006), and Scopus (since 2012) greatly expanded its visibility and influence.^2^ ^,^ ^3^ This global recognition culminated recently in the remarkable achievement of an impact factor of 3.0,^2^ ^,^ ^3^ a testament to the growing relevance and excellence of the research published within its pages. This milestone places the JBP among the most respected journals in respiratory medicine worldwide and reflects both the quality of Brazilian science and the dedication of its editorial leadership.^4^

Recent years have tested and confirmed the journal’s vital role. During public health emergencies-from the H1N1 influenza epidemic to the COVID-19 pandemic-the JBP offered timely, free access to life-saving research, national guidelines, and new clinical insights, thus becoming an essential resource for practitioners and policymakers alike.^5^ ^-^ ^7^

The trajectory of the JBP is inseparable from that of the SBPT and the broader respiratory medicine community in Brazil. From the early days of typewritten manuscripts to its present era as a fully digital, internationally recognized scientific journal, the JBP has been defined by rigor, independence, and a profound commitment to the advancement of respiratory health.^1^ ^-^ ^3^

As we commemorate five decades of achievements, we honor everyone who has contributed to this legacy: the editors-in-chief, associate editors, peer reviewers, authors, dedicated readers, and SBPT members. We also remember and thank the founders who imagined and built this platform, paving the way for future generations.

Looking ahead, we reaffirm our mission: to foster scientific excellence, promote lifelong education, and advocate for stronger respiratory health policies. The challenges that await us-emerging diseases, environmental threats, and barriers to access-only strengthen our resolve to innovate and collaborate, led by the strong and respected voice of our journal.

May the JBP continue to illuminate our path, strengthen professional bonds, and inspire pulmonologists throughout Brazil and the world.

Congratulations to all who are part of this remarkable journey!
